# *cis*-1-Ethyl-4,4,6,8-tetra­methyl-2-tosyl-2,3,3a,4,6,7,8,9-octa­hydro-1*H*-pyrrolo[3′,4′:3,4]pyrano[6,5-*d*]pyrimidine-7,9-dione

**DOI:** 10.1107/S1600536809026361

**Published:** 2009-07-15

**Authors:** K. Chinnakali, D. Sudha, M. Jayagobi, R. Raghunathan, Hoong-Kun Fun

**Affiliations:** a Department of Physics, Anna University Chennai, Chennai 600 025, India; b Department of Organic Chemistry, University of Madras, Guindy Campus, Chennai 600 025, India; c X-ray Crystallography Unit, School of Physics, Universiti Sains Malaysia, 11800 USM, Penang, Malaysia

## Abstract

In the title compound, C_22_H_29_N_3_O_5_S, the pyrrolidine ring is *cis*-fused to the dihydro­pyran ring. The pyrrolidine and dihydro­pyran rings adopt twist and half-chair conformations, respectively. The mol­ecule is in a folded conformation; the sulfonyl-bound benzene ring lies over the pyrimidine­dione ring, with a weak π–π inter­action [centroid–centroid distance = 3.6147 (4) Å]. A weak intra­molecular C—H⋯O hydrogen bond generates an *S*(6) ring motif. In the crystal, molecules are linked into a three-dimensional network by C—H⋯O hydrogen bonds.

## Related literature

For the *trans* isomer, see: Chinnakali *et al.* (2007 ▶). For the biological activity of pyran­opyrimidine derivatives, see: Abdel Fattah *et al.* (2004 ▶); Bedair *et al.* (2000 ▶, 2001 ▶); Eid *et al.* (2004 ▶); Shamroukh *et al.* (2007 ▶). For ring puckering parameters, see: Cremer & Pople (1975 ▶). For asymmetry parameters, see: Duax *et al.* (1976 ▶).
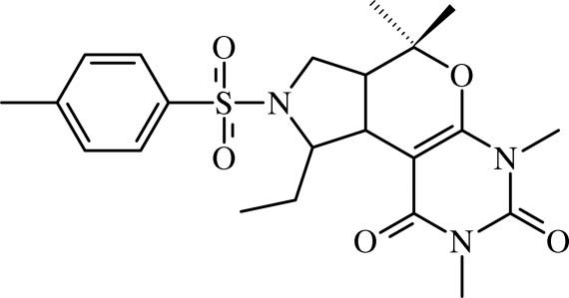


## Experimental

### 

#### Crystal data

C_22_H_29_N_3_O_5_S
*M* *_r_* = 447.54
Monoclinic, 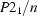

*a* = 13.2140 (2) Å
*b* = 9.5681 (2) Å
*c* = 16.8256 (3) Å
β = 98.946 (1)°
*V* = 2101.43 (7) Å^3^
*Z* = 4
Mo *K*α radiation
μ = 0.20 mm^−1^
*T* = 100 K
0.59 × 0.46 × 0.29 mm

#### Data collection

Bruker SMART APEXII CCD area-detector diffractometer
Absorption correction: multi-scan (*SADABS*; Bruker, 2005 ▶) *T*_min_ = 0.864, *T*_max_ = 0.945
92335 measured reflections
10993 independent reflections
9848 reflections with *I* > 2σ(*I*)
*R*_int_ = 0.026

#### Refinement

*R*[*F* ^2^ > 2σ(*F* ^2^)] = 0.033
*wR*(*F* ^2^) = 0.105
*S* = 1.08
10993 reflections
286 parameters
H-atom parameters constrained
Δρ_max_ = 0.60 e Å^−3^
Δρ_min_ = −0.43 e Å^−3^

### 

Data collection: *APEX2* (Bruker, 2005 ▶); cell refinement: *SAINT* (Bruker, 2005 ▶); data reduction: *SAINT*; program(s) used to solve structure: *SHELXTL* (Sheldrick, 2008 ▶); program(s) used to refine structure: *SHELXTL*; molecular graphics: *SHELXTL*; software used to prepare material for publication: *SHELXTL* and *PLATON* (Spek, 2009 ▶).

## Supplementary Material

Crystal structure: contains datablocks global, I. DOI: 10.1107/S1600536809026361/wn2336sup1.cif

Structure factors: contains datablocks I. DOI: 10.1107/S1600536809026361/wn2336Isup2.hkl

Additional supplementary materials:  crystallographic information; 3D view; checkCIF report

## Figures and Tables

**Table 1:** Hydrogen-bond geometry (Å, °)

*D*—H⋯*A*	*D*—H	H⋯*A*	*D*⋯*A*	*D*—H⋯*A*
C4—H4⋯O5	0.98	2.43	3.0422 (8)	120
C1—H1*B*⋯O4^i^	0.97	2.54	3.5072 (8)	177
C16—H16*B*⋯O5^ii^	0.96	2.57	3.3914 (8)	144
C19—H19*C*⋯O1^iii^	0.96	2.52	3.4006 (9)	153
C20—H20*C*⋯O2^iv^	0.96	2.51	3.2335 (8)	132

Symmetry codes: (i) 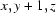; (ii) 
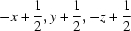
; (iii) 
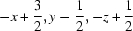
; (iv) 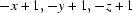.

**Fig. 1.**
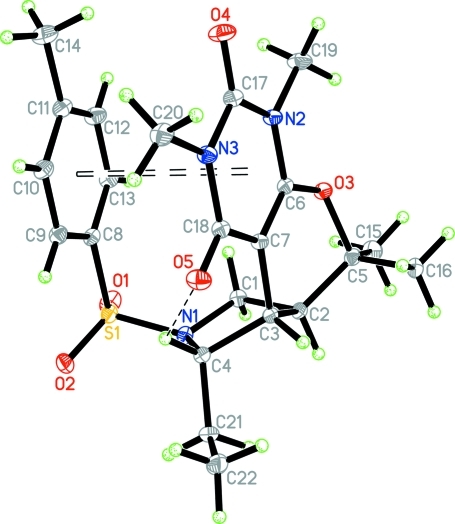
The molecular structure of the title compound. Displacement ellipsoids are drawn at the 50% probability level. Hydrogen atoms are shown as spheres of arbitrary radius. The dashed open line indicates a π-π interaction and the dashed line indicates a hydrogen bond.

**Fig. 2.**
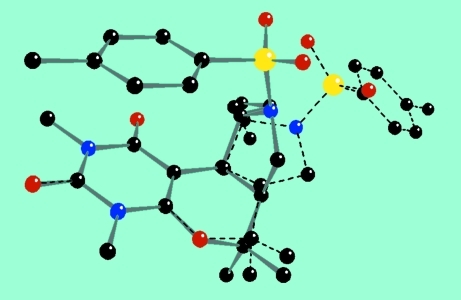
Fit of the title molecule (solid lines) on molecule A (dashed lines) of the trans isomer. H atoms have been omitted for clarity.

**Fig. 3.**
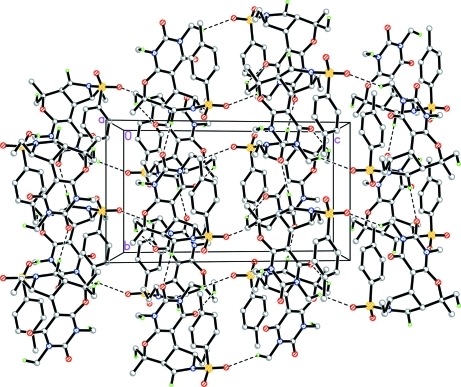
Crystal packing of the title compound. Hydrogen bonds are shown as dashed lines. For the sake of clarity, H atoms not involved in the interactions have been omitted.

## supplementary crystallographic information

### Comment

Pyranopyrimidine derivatives exhibit antiviral (Shamroukh *et al.*, 2007) and antimicrobial activities (Bedair *et al.*, 2000, 2001; Eid *et al.*, 2004; Abdel Fattah *et al.*, 2004). Previously, we have reported the crystal structure of *trans*-1-ethyl-4,4,6,8-tetramethyl- 2-tosyl-2,3,3a,4,6,7,8,9-octahydro-1*H*-pyrrolo[3,4-*c*]pyrano[6,5-*d*]pyrimidine-7,9-dione (Chinnakali *et al.*, 2007). Now we report the crystal structure of the title compound, a *cis* isomer.

In the title *cis* isomer, the pyrrolidine ring (N1/C1—C4) adopts a twist conformation compared to envelope conformations in the two independent molecules of the *trans* isomer. The relevant asymmetry parameter (Duax *et al.*, 1976) ΔC_2_[C2—C3] is 6.01 (6)°, and Cremer & Pople (1975) puckering parameters Q and φ are 0.3823 (7) Å and 264.96 (10)°, respectively. The dihydropyran ring adopts a half-chair conformation, with the local twofold axis passing through the midpoint of the C2—C5 and C6—C7 bonds. The asymmetry parameter ΔC_2_[C2–C5] is 4.33 (7)° and the puckering parameters Q, θ and φ are 0.4800 (7) Å, 127.48 (7)° and 267.38 (9)°, respectively. In both independent molecules of the *trans* isomer, the dihydropyran ring adopts an envelope conformation. The tosyl group is equatorially attached to the pyrrolidine ring, whereas the ethyl group is axially attached. The sulfonyl group has a distorted tetrahedral geometry. The pyrrolidine ring is *cis*-fused to the dihydropyran ring. The dihedral angle between the pyrimidine and benzene rings is 10.84 (3)°. The corresponding bond lengths and angles in the two isomers agree with each other.

A superposition of non-H atoms in the dimethyl pyrimidine-7,9-dione unit of the *cis* isomer (title molecule) and molecule A of the *trans* isomer (Chinnakali *et al.*, 2007) (Fig. 2) shows that the overall conformations of these isomers are different. The *trans *-fusion results in an extended ring system, with the tosyl group bending away from the fused ring system. In the *cis*-isomer, the molecule is in a folded conformation, with the sulfonyl-bound benzene ring lying over the pyrimidinedione ring. As a result of the folded conformation, the benzene and pyrimidinedione rings of the *cis* isomer are placed one over the other with weak π-π interactions (centroid-centroid distance = 3.6147 (4) Å).

A weak intramolecular C4—H4···O5 hydrogen bond generates an S(6) ring motif. The molecules exist as a C—H···O hydrogen-bonded dimer, generating a ring of graph-set motif *R*^2^_2_(20). The dimers are linked into a three-dimensional network by C—H···O hydrogen bonds (Fig. 3).

### Experimental

To a solution of barbituric acid (1 mmol) in toluene (20 ml) the corresponding 2-(*N*-prenyl-*N*-tosylamino]acetaldehyde (1 mmol) and a catalytic amount of the base ethylenediamine-*N*,*N*'-diacetate (EDDA) were added and the reaction mixture was refluxed for 12 h. After completion of the reaction, the solvent was evaporated under reduced pressure and the crude product was chromatographed using a hexane-ethyl acetate (8:2 *v*/*v*) mixture to obtain the title compound. The compound was recrystallized from ethyl acetate solution by slow evaporation.

### Refinement

All H atoms were positioned geometrically and allowed to ride on their parent atoms, with C—H = 0.93–0.98 Å. The *U*_iso_ values were set equal to 1.5*U*_eq_ of the carrier atom for methyl H atoms and 1.2*U*_eq_ for the remaining H atoms. A rotating group model was used for the methyl groups.

### Crystal data

**Table:**

C_22_H_29_N_3_O_5_S	*F*(000) = 952
*M**_r_* = 447.54	*D*_x_ = 1.415 Mg m^−^^3^
Monoclinic, *P*2_1_/*n*	Mo *K*α radiation, λ = 0.71073 Å
Hall symbol: -P 2yn	Cell parameters from 9302 reflections
*a* = 13.2140 (2) Å	θ = 2.5–40.9°
*b* = 9.5681 (2) Å	µ = 0.20 mm^−^^1^
*c* = 16.8256 (3) Å	*T* = 100 K
β = 98.946 (1)°	Block, colourless
*V* = 2101.43 (7) Å^3^	0.59 × 0.46 × 0.29 mm
*Z* = 4	

### Data collection

**Table:**

Bruker SMART APEXII CCD area-detector diffractometer	10993 independent reflections
Radiation source: fine-focus sealed tube	9848 reflections with *I* > 2σ(*I*)
graphite	*R*_int_ = 0.026
φ and ω scans	θ_max_ = 37.5°, θ_min_ = 1.8°
Absorption correction: multi-scan (*SADABS*; Bruker, 2005)	*h* = −22→20
*T*_min_ = 0.864, *T*_max_ = 0.945	*k* = −16→16
92335 measured reflections	*l* = −28→28

### Refinement

**Table:**

Refinement on *F*^2^	Primary atom site location: structure-invariant direct methods
Least-squares matrix: full	Secondary atom site location: difference Fourier map
*R*[*F*^2^ > 2σ(*F*^2^)] = 0.033	Hydrogen site location: inferred from neighbouring sites
*wR*(*F*^2^) = 0.105	H-atom parameters constrained
*S* = 1.08	*w* = 1/[σ^2^(*F*_o_^2^) + (0.0595*P*)^2^ + 0.4609*P*] where *P* = (*F*_o_^2^ + 2*F*_c_^2^)/3
10993 reflections	(Δ/σ)_max_ = 0.002
286 parameters	Δρ_max_ = 0.60 e Å^−^^3^
0 restraints	Δρ_min_ = −0.43 e Å^−^^3^

### Special details

**Table:**

Geometry. All e.s.d.'s (except the e.s.d. in the dihedral angle between two l.s. planes) are estimated using the full covariance matrix. The cell e.s.d.'s are taken into account individually in the estimation of e.s.d.'s in distances, angles and torsion angles; correlations between e.s.d.'s in cell parameters are only used when they are defined by crystal symmetry. An approximate (isotropic) treatment of cell e.s.d.'s is used for estimating e.s.d.'s involving l.s. planes.
Refinement. Refinement of *F*^2^ against ALL reflections. The weighted *R*-factor *wR* and goodness of fit *S* are based on *F*^2^, conventional *R*-factors *R* are based on *F*, with *F* set to zero for negative *F*^2^. The threshold expression of *F*^2^ > σ(*F*^2^) is used only for calculating *R*-factors(gt) *etc*. and is not relevant to the choice of reflections for refinement. *R*-factors based on *F*^2^ are statistically about twice as large as those based on *F*, and *R*- factors based on ALL data will be even larger.

### Fractional atomic coordinates and isotropic or equivalent isotropic displacement parameters (Å^2^)

**Table:**

	*x*	*y*	*z*	*U*_iso_*/*U*_eq_	
S1	0.583987 (12)	0.842927 (15)	0.406869 (9)	0.01234 (4)	
O1	0.65851 (4)	0.94222 (5)	0.38823 (4)	0.01920 (10)	
O2	0.54783 (5)	0.84656 (6)	0.48296 (3)	0.01802 (10)	
O3	0.48049 (4)	0.66605 (5)	0.12573 (3)	0.01221 (8)	
O4	0.56281 (5)	0.22291 (5)	0.20707 (4)	0.01945 (10)	
O5	0.34489 (4)	0.46219 (5)	0.34319 (3)	0.01542 (9)	
N1	0.48436 (4)	0.86053 (6)	0.33745 (3)	0.01170 (8)	
N2	0.52189 (4)	0.44704 (5)	0.16924 (3)	0.01178 (8)	
N3	0.45765 (4)	0.34362 (5)	0.27803 (3)	0.01201 (9)	
C1	0.50142 (5)	0.87879 (6)	0.25331 (4)	0.01246 (9)	
H1A	0.5568	0.8197	0.2414	0.015*	
H1B	0.5170	0.9753	0.2425	0.015*	
C2	0.39850 (5)	0.83406 (6)	0.20456 (4)	0.01116 (9)	
H2	0.3507	0.9129	0.2011	0.013*	
C3	0.36105 (4)	0.71839 (6)	0.25639 (3)	0.01025 (9)	
H3	0.2865	0.7081	0.2437	0.012*	
C4	0.39216 (4)	0.77336 (6)	0.34312 (4)	0.01094 (9)	
H4	0.4110	0.6948	0.3798	0.013*	
C5	0.40701 (5)	0.78390 (6)	0.11965 (4)	0.01210 (9)	
C6	0.46937 (4)	0.56814 (6)	0.18057 (3)	0.01023 (9)	
C7	0.41350 (4)	0.58284 (6)	0.24165 (3)	0.01009 (9)	
C8	0.63400 (5)	0.67386 (6)	0.39643 (4)	0.01179 (9)	
C9	0.60216 (5)	0.56445 (7)	0.44136 (4)	0.01306 (10)	
H9	0.5562	0.5809	0.4769	0.016*	
C10	0.63973 (5)	0.43040 (7)	0.43263 (4)	0.01394 (10)	
H10	0.6177	0.3572	0.4620	0.017*	
C11	0.71018 (5)	0.40403 (7)	0.38037 (4)	0.01433 (10)	
C12	0.74167 (5)	0.51562 (7)	0.33623 (4)	0.01645 (11)	
H12	0.7884	0.4996	0.3012	0.020*	
C13	0.70416 (5)	0.64999 (7)	0.34392 (4)	0.01486 (10)	
H13	0.7257	0.7233	0.3143	0.018*	
C14	0.75187 (6)	0.25884 (7)	0.37293 (5)	0.02037 (12)	
H14A	0.7406	0.2040	0.4185	0.031*	
H14B	0.8240	0.2640	0.3709	0.031*	
H14C	0.7175	0.2160	0.3246	0.031*	
C15	0.45325 (6)	0.89403 (7)	0.07111 (4)	0.01763 (11)	
H15A	0.4595	0.8570	0.0191	0.026*	
H15B	0.5198	0.9198	0.0987	0.026*	
H15C	0.4097	0.9749	0.0649	0.026*	
C16	0.30475 (5)	0.73203 (7)	0.07499 (4)	0.01565 (10)	
H16A	0.3128	0.7040	0.0216	0.023*	
H16B	0.2550	0.8057	0.0721	0.023*	
H16C	0.2820	0.6537	0.1031	0.023*	
C17	0.51744 (5)	0.33126 (6)	0.21815 (4)	0.01279 (9)	
C18	0.40139 (5)	0.46334 (6)	0.29140 (4)	0.01094 (9)	
C19	0.58284 (6)	0.43377 (7)	0.10385 (4)	0.01794 (12)	
H19A	0.5632	0.5055	0.0646	0.027*	
H19B	0.5709	0.3437	0.0790	0.027*	
H19C	0.6542	0.4433	0.1252	0.027*	
C20	0.45052 (6)	0.22000 (7)	0.32843 (4)	0.01626 (11)	
H20A	0.5176	0.1809	0.3441	0.024*	
H20B	0.4067	0.1518	0.2986	0.024*	
H20C	0.4226	0.2464	0.3756	0.024*	
C21	0.30950 (5)	0.86157 (7)	0.37333 (4)	0.01587 (11)	
H21A	0.3397	0.9115	0.4214	0.019*	
H21B	0.2841	0.9303	0.3327	0.019*	
C22	0.21975 (5)	0.77395 (9)	0.39250 (4)	0.02000 (13)	
H22A	0.1715	0.8334	0.4133	0.030*	
H22B	0.2445	0.7044	0.4319	0.030*	
H22C	0.1867	0.7290	0.3444	0.030*	

### Atomic displacement parameters (Å^2^)

**Table:**

	*U*^11^	*U*^22^	*U*^33^	*U*^12^	*U*^13^	*U*^23^
S1	0.01271 (7)	0.00984 (6)	0.01338 (7)	−0.00042 (4)	−0.00136 (5)	−0.00091 (4)
O1	0.0160 (2)	0.01258 (19)	0.0272 (3)	−0.00482 (16)	−0.00210 (18)	0.00116 (17)
O2	0.0232 (2)	0.0180 (2)	0.01203 (19)	0.00396 (17)	−0.00003 (17)	−0.00289 (15)
O3	0.01293 (18)	0.01207 (17)	0.01232 (18)	0.00183 (14)	0.00415 (14)	0.00262 (13)
O4	0.0223 (2)	0.01282 (19)	0.0246 (2)	0.00660 (17)	0.00808 (19)	0.00096 (17)
O5	0.0171 (2)	0.01400 (19)	0.0170 (2)	−0.00069 (15)	0.00842 (16)	0.00159 (15)
N1	0.0117 (2)	0.01097 (18)	0.01199 (19)	−0.00025 (15)	0.00035 (15)	0.00063 (15)
N2	0.0119 (2)	0.01108 (19)	0.0130 (2)	0.00168 (15)	0.00410 (15)	0.00013 (15)
N3	0.0144 (2)	0.00921 (18)	0.0128 (2)	0.00074 (15)	0.00319 (16)	0.00089 (14)
C1	0.0135 (2)	0.0111 (2)	0.0128 (2)	−0.00121 (17)	0.00190 (18)	0.00083 (17)
C2	0.0116 (2)	0.0103 (2)	0.0113 (2)	0.00114 (16)	0.00109 (17)	0.00142 (16)
C3	0.0096 (2)	0.0106 (2)	0.0106 (2)	0.00110 (16)	0.00170 (16)	0.00018 (16)
C4	0.0104 (2)	0.0110 (2)	0.0113 (2)	0.00091 (16)	0.00145 (16)	−0.00006 (16)
C5	0.0126 (2)	0.0122 (2)	0.0115 (2)	0.00189 (17)	0.00177 (17)	0.00231 (17)
C6	0.0096 (2)	0.00993 (19)	0.0111 (2)	0.00013 (16)	0.00149 (16)	0.00011 (16)
C7	0.0100 (2)	0.00933 (19)	0.0112 (2)	0.00002 (16)	0.00243 (16)	0.00026 (16)
C8	0.0106 (2)	0.0112 (2)	0.0131 (2)	0.00007 (16)	0.00020 (17)	0.00090 (17)
C9	0.0124 (2)	0.0130 (2)	0.0139 (2)	0.00026 (17)	0.00232 (18)	0.00160 (17)
C10	0.0134 (2)	0.0122 (2)	0.0162 (2)	0.00041 (18)	0.00204 (18)	0.00224 (18)
C11	0.0130 (2)	0.0128 (2)	0.0168 (2)	0.00140 (18)	0.00108 (19)	0.00025 (19)
C12	0.0151 (3)	0.0158 (2)	0.0196 (3)	0.0021 (2)	0.0060 (2)	0.0020 (2)
C13	0.0136 (2)	0.0139 (2)	0.0176 (3)	0.00009 (18)	0.0042 (2)	0.00289 (19)
C14	0.0212 (3)	0.0142 (2)	0.0264 (3)	0.0046 (2)	0.0058 (2)	0.0002 (2)
C15	0.0218 (3)	0.0159 (3)	0.0160 (2)	0.0006 (2)	0.0054 (2)	0.0053 (2)
C16	0.0140 (2)	0.0202 (3)	0.0120 (2)	0.0018 (2)	−0.00014 (18)	−0.00042 (19)
C17	0.0128 (2)	0.0109 (2)	0.0148 (2)	0.00118 (17)	0.00253 (18)	−0.00002 (17)
C18	0.0108 (2)	0.0098 (2)	0.0122 (2)	−0.00062 (16)	0.00179 (16)	−0.00023 (16)
C19	0.0202 (3)	0.0163 (3)	0.0197 (3)	0.0048 (2)	0.0108 (2)	0.0017 (2)
C20	0.0205 (3)	0.0109 (2)	0.0177 (3)	0.00067 (19)	0.0043 (2)	0.00368 (19)
C21	0.0147 (2)	0.0179 (3)	0.0153 (2)	0.0050 (2)	0.00301 (19)	−0.0024 (2)
C22	0.0132 (3)	0.0310 (4)	0.0163 (3)	0.0044 (2)	0.0041 (2)	0.0025 (2)

### Geometric parameters (Å, °)

**Table:**

S1—O2	1.4348 (6)	C8—C13	1.3954 (9)
S1—O1	1.4376 (6)	C9—C10	1.3914 (9)
S1—N1	1.6271 (6)	C9—H9	0.93
S1—C8	1.7665 (6)	C10—C11	1.3992 (9)
O3—C6	1.3391 (7)	C10—H10	0.93
O3—C5	1.4811 (8)	C11—C12	1.4003 (10)
O4—C17	1.2262 (8)	C11—C14	1.5068 (9)
O5—C18	1.2326 (7)	C12—C13	1.3912 (9)
N1—C1	1.4783 (8)	C12—H12	0.93
N1—C4	1.4917 (8)	C13—H13	0.93
N2—C6	1.3790 (8)	C14—H14A	0.96
N2—C17	1.3867 (8)	C14—H14B	0.96
N2—C19	1.4666 (8)	C14—H14C	0.96
N3—C17	1.3787 (8)	C15—H15A	0.96
N3—C18	1.4026 (8)	C15—H15B	0.96
N3—C20	1.4669 (8)	C15—H15C	0.96
C1—C2	1.5357 (9)	C16—H16A	0.96
C1—H1A	0.97	C16—H16B	0.96
C1—H1B	0.97	C16—H16C	0.96
C2—C5	1.5277 (9)	C19—H19A	0.96
C2—C3	1.5377 (8)	C19—H19B	0.96
C2—H2	0.98	C19—H19C	0.96
C3—C7	1.5091 (8)	C20—H20A	0.96
C3—C4	1.5455 (8)	C20—H20B	0.96
C3—H3	0.98	C20—H20C	0.96
C4—C21	1.5285 (9)	C21—C22	1.5273 (11)
C4—H4	0.98	C21—H21A	0.97
C5—C15	1.5185 (9)	C21—H21B	0.97
C5—C16	1.5236 (9)	C22—H22A	0.96
C6—C7	1.3627 (8)	C22—H22B	0.96
C7—C18	1.4405 (8)	C22—H22C	0.96
C8—C9	1.3940 (9)		
			
O2—S1—O1	120.86 (3)	C9—C10—H10	119.5
O2—S1—N1	107.07 (3)	C11—C10—H10	119.5
O1—S1—N1	106.28 (3)	C10—C11—C12	118.52 (6)
O2—S1—C8	107.01 (3)	C10—C11—C14	120.40 (6)
O1—S1—C8	107.69 (3)	C12—C11—C14	121.08 (6)
N1—S1—C8	107.27 (3)	C13—C12—C11	121.05 (6)
C6—O3—C5	116.05 (5)	C13—C12—H12	119.5
C1—N1—C4	112.02 (5)	C11—C12—H12	119.5
C1—N1—S1	118.22 (4)	C12—C13—C8	119.44 (6)
C4—N1—S1	118.29 (4)	C12—C13—H13	120.3
C6—N2—C17	121.36 (5)	C8—C13—H13	120.3
C6—N2—C19	121.55 (5)	C11—C14—H14A	109.5
C17—N2—C19	117.06 (5)	C11—C14—H14B	109.5
C17—N3—C18	124.43 (5)	H14A—C14—H14B	109.5
C17—N3—C20	116.67 (5)	C11—C14—H14C	109.5
C18—N3—C20	118.85 (5)	H14A—C14—H14C	109.5
N1—C1—C2	102.97 (5)	H14B—C14—H14C	109.5
N1—C1—H1A	111.2	C5—C15—H15A	109.5
C2—C1—H1A	111.2	C5—C15—H15B	109.5
N1—C1—H1B	111.2	H15A—C15—H15B	109.5
C2—C1—H1B	111.2	C5—C15—H15C	109.5
H1A—C1—H1B	109.1	H15A—C15—H15C	109.5
C5—C2—C1	113.53 (5)	H15B—C15—H15C	109.5
C5—C2—C3	112.41 (5)	C5—C16—H16A	109.5
C1—C2—C3	103.53 (5)	C5—C16—H16B	109.5
C5—C2—H2	109.1	H16A—C16—H16B	109.5
C1—C2—H2	109.1	C5—C16—H16C	109.5
C3—C2—H2	109.1	H16A—C16—H16C	109.5
C7—C3—C2	109.20 (5)	H16B—C16—H16C	109.5
C7—C3—C4	112.71 (5)	O4—C17—N3	122.21 (6)
C2—C3—C4	103.22 (5)	O4—C17—N2	121.22 (6)
C7—C3—H3	110.5	N3—C17—N2	116.56 (5)
C2—C3—H3	110.5	O5—C18—N3	120.03 (5)
C4—C3—H3	110.5	O5—C18—C7	123.51 (5)
N1—C4—C21	110.21 (5)	N3—C18—C7	116.46 (5)
N1—C4—C3	103.29 (5)	N2—C19—H19A	109.5
C21—C4—C3	113.77 (5)	N2—C19—H19B	109.5
N1—C4—H4	109.8	H19A—C19—H19B	109.5
C21—C4—H4	109.8	N2—C19—H19C	109.5
C3—C4—H4	109.8	H19A—C19—H19C	109.5
O3—C5—C15	104.54 (5)	H19B—C19—H19C	109.5
O3—C5—C16	107.92 (5)	N3—C20—H20A	109.5
C15—C5—C16	111.16 (5)	N3—C20—H20B	109.5
O3—C5—C2	108.51 (5)	H20A—C20—H20B	109.5
C15—C5—C2	112.29 (5)	N3—C20—H20C	109.5
C16—C5—C2	112.02 (5)	H20A—C20—H20C	109.5
O3—C6—C7	125.30 (5)	H20B—C20—H20C	109.5
O3—C6—N2	112.40 (5)	C22—C21—C4	112.69 (6)
C7—C6—N2	122.30 (5)	C22—C21—H21A	109.1
C6—C7—C18	118.58 (5)	C4—C21—H21A	109.1
C6—C7—C3	121.95 (5)	C22—C21—H21B	109.1
C18—C7—C3	119.43 (5)	C4—C21—H21B	109.1
C9—C8—C13	120.45 (6)	H21A—C21—H21B	107.8
C9—C8—S1	118.99 (5)	C21—C22—H22A	109.5
C13—C8—S1	120.56 (5)	C21—C22—H22B	109.5
C10—C9—C8	119.49 (6)	H22A—C22—H22B	109.5
C10—C9—H9	120.3	C21—C22—H22C	109.5
C8—C9—H9	120.3	H22A—C22—H22C	109.5
C9—C10—C11	121.05 (6)	H22B—C22—H22C	109.5
			
O2—S1—N1—C1	172.18 (4)	N2—C6—C7—C3	176.56 (5)
O1—S1—N1—C1	41.74 (5)	C2—C3—C7—C6	−10.74 (8)
C8—S1—N1—C1	−73.25 (5)	C4—C3—C7—C6	−124.84 (6)
O2—S1—N1—C4	−47.28 (5)	C2—C3—C7—C18	171.67 (5)
O1—S1—N1—C4	−177.72 (5)	C4—C3—C7—C18	57.57 (7)
C8—S1—N1—C4	67.29 (5)	O2—S1—C8—C9	21.57 (6)
C4—N1—C1—C2	15.62 (6)	O1—S1—C8—C9	152.92 (5)
S1—N1—C1—C2	158.49 (4)	N1—S1—C8—C9	−93.05 (5)
N1—C1—C2—C5	−155.54 (5)	O2—S1—C8—C13	−158.55 (5)
N1—C1—C2—C3	−33.37 (6)	O1—S1—C8—C13	−27.20 (6)
C5—C2—C3—C7	41.74 (6)	N1—S1—C8—C13	86.83 (6)
C1—C2—C3—C7	−81.18 (5)	C13—C8—C9—C10	−0.94 (9)
C5—C2—C3—C4	161.86 (5)	S1—C8—C9—C10	178.93 (5)
C1—C2—C3—C4	38.94 (6)	C8—C9—C10—C11	0.98 (10)
C1—N1—C4—C21	−113.51 (6)	C9—C10—C11—C12	−0.55 (10)
S1—N1—C4—C21	103.65 (5)	C9—C10—C11—C14	178.68 (6)
C1—N1—C4—C3	8.38 (6)	C10—C11—C12—C13	0.07 (10)
S1—N1—C4—C3	−134.46 (4)	C14—C11—C12—C13	−179.16 (7)
C7—C3—C4—N1	88.77 (5)	C11—C12—C13—C8	−0.04 (11)
C2—C3—C4—N1	−28.91 (6)	C9—C8—C13—C12	0.48 (10)
C7—C3—C4—C21	−151.76 (5)	S1—C8—C13—C12	−179.40 (5)
C2—C3—C4—C21	90.56 (6)	C18—N3—C17—O4	−177.65 (6)
C6—O3—C5—C15	165.61 (5)	C20—N3—C17—O4	−0.07 (10)
C6—O3—C5—C16	−75.98 (6)	C18—N3—C17—N2	0.78 (9)
C6—O3—C5—C2	45.61 (7)	C20—N3—C17—N2	178.35 (6)
C1—C2—C5—O3	57.44 (6)	C6—N2—C17—O4	178.74 (6)
C3—C2—C5—O3	−59.67 (6)	C19—N2—C17—O4	0.17 (10)
C1—C2—C5—C15	−57.61 (7)	C6—N2—C17—N3	0.30 (9)
C3—C2—C5—C15	−174.72 (5)	C19—N2—C17—N3	−178.27 (6)
C1—C2—C5—C16	176.47 (5)	C17—N3—C18—O5	175.47 (6)
C3—C2—C5—C16	59.37 (7)	C20—N3—C18—O5	−2.06 (9)
C5—O3—C6—C7	−15.49 (8)	C17—N3—C18—C7	−4.13 (9)
C5—O3—C6—N2	164.75 (5)	C20—N3—C18—C7	178.34 (6)
C17—N2—C6—O3	−177.85 (5)	C6—C7—C18—O5	−173.12 (6)
C19—N2—C6—O3	0.65 (8)	C3—C7—C18—O5	4.56 (9)
C17—N2—C6—C7	2.38 (9)	C6—C7—C18—N3	6.47 (8)
C19—N2—C6—C7	−179.12 (6)	C3—C7—C18—N3	−175.86 (5)
O3—C6—C7—C18	174.43 (6)	N1—C4—C21—C22	−171.35 (5)
N2—C6—C7—C18	−5.82 (9)	C3—C4—C21—C22	73.20 (7)
O3—C6—C7—C3	−3.19 (9)		

### Hydrogen-bond geometry (Å, °)

**Table:**

*D*—H···*A*	*D*—H	H···*A*	*D*···*A*	*D*—H···*A*
C4—H4···O5	0.98	2.43	3.0422 (8)	120
C1—H1B···O4^i^	0.97	2.54	3.5072 (8)	177
C16—H16B···O5^ii^	0.96	2.57	3.3914 (8)	144
C19—H19C···O1^iii^	0.96	2.52	3.4006 (9)	153
C20—H20C···O2^iv^	0.96	2.51	3.2335 (8)	132

Symmetry codes: (i) *x*, *y*+1, *z*; (ii) −*x*+1/2, *y*+1/2, −*z*+1/2; (iii) −*x*+3/2, *y*−1/2, −*z*+1/2; (iv) −*x*+1, −*y*+1, −*z*+1.
